# Femoral Reconstruction Using External Fixation

**DOI:** 10.4061/2011/967186

**Published:** 2011-03-15

**Authors:** Yevgeniy Palatnik, S. Robert Rozbruch

**Affiliations:** Limb Lengthening and Deformity Service, Hospital for Special Surgery, Weill Medical College of Cornell University, New York, NY 10065, USA

## Abstract

*Background*. The use of an external fixator for the purpose of distraction osteogenesis has been applied to a wide range of orthopedic problems caused by such diverse etiologies as congenital disease, metabolic conditions, infections, traumatic injuries, and congenital short stature. The purpose of this study was to analyze our experience of utilizing this method in patients undergoing a variety of orthopedic procedures of the femur. *Methods*. We retrospectively reviewed our experience of using external fixation for femoral reconstruction. Three subgroups were defined based on the primary reconstruction goal lengthening, deformity correction, and repair of nonunion/bone defect. Factors such as leg length discrepancy (LLD), limb alignment, and external fixation time and complications were evaluated for the entire group and the 3 subgroups. *Results*. There was substantial improvement in the overall LLD, femoral length discrepancy, and limb alignment as measured by mechanical axis deviation (MAD) and lateral distal femoral angle (LDFA) for the entire group as well as the subgroups. *Conclusions*. The Ilizarov external fixator allows for decreased surgical exposure and preservation of blood supply to bone, avoidance of bone grafting and internal fixation, and simultaneous lengthening and deformity correction, making it a very useful technique for femoral reconstruction.

## 1. Introduction

Distraction osteogenesis with the use of an external fixator has been reported in multiple studies to have been used successfully for the treatment of a wide range of orthopedic problems including limb-length discrepancy [1–5], nonunions and bone defects [6–13], and deformity correction [2, 3, 14–20] caused by congenital disease [3, 14, 17], metabolic conditions [16], infections [6, 8–11, 21], traumatic injuries [7, 15], and congenital short stature [1, 4]. 

This method carries with it several advantages including minimal blood loss and limited surgical exposure. These factors make this approach especially applicable to patients with poor skin and/or pre-existent soft-tissue or bone infections. Femoral reconstruction using the Ilizarov method and an external fixator offers the surgeon a comprehensive approach to a wide variety of femur pathologies due to the ability to perform simultaneous limb lengthening as well as allowing correction of deformities and bone transport [10, 15, 22]. In most cases bone grafting and internal fixation can be avoided which is particularly beneficial when there is either active infection or history thereof [1]. 

In this study we retrospectively analyzed our aggregate experience of using the Ilizarov method and external fixation for the purposes of femoral lengthening, deformity correction, and repair of nonunions and bone defects.

## 2. Methods

Patients who underwent surgeries of the femur utilizing external fixation in our practice were identified from an IRB approved limb reconstruction registry. Overall, the results for 47 femora in 43 patients were retrospectively reviewed. In analyzing the patient population in this study, 3 separate patient groups were identified based on the primary goal for using external fixation. These groups included lengthening (performed in 21 patients, 23 femora), deformity correction (12 patients, 14 femora), and repair of nonunion/bone defect (10 patients, 10 femora). Both quantitative and qualitative characteristics of patients were recorded. These were in turn examined separately in the setting of separate patient groups. 

Basic demographic data for each patient (sex, age at time of surgery) was recorded. Diagnoses and their etiologies were noted and were used to stratify patients into the 3 groups. Pre-existing medical conditions which may interfere with bone healing were noted. These included rheumatoid arthritis, diabetes mellitus, osteomyelitis, and neuropathy. Presence of varus, valgus, procurvatum, recurvatum, internal and external rotation deformities was noted. The extent of the former two was measured by mechanical axis deviation (MAD) and lateral distal femoral angle (LDFA). Varus or medial MAD and valgus or lateral MAD were recorded and averaged separately in order to most accurately portray the limb alignment. Changes in the range of motion of the knee joint were compared before and after surgery. The limb length discrepancy before and after surgery and the amount of lengthening achieved were also recorded. The amount of time that patients spent in the frame was broken down into the distraction time and the consolidation time. External fixation index (EFI), which is the number of months the patients wore the frame per cm of lengthening done, was also recorded. The utilization of bone grafting and/or bone stimulator was noted. We also analyzed the incidence of complications, both those that did and those that did not require additional surgical interventions.

## 3. Results

For the entire group, the average follow-up was 42 months (range, 9–77). Average time in frame was 6 months (range, 1–18). Varus and valgus deformity was improved with surgery as measured by LDFA and MAD (Table 1). Bone lengthening of 5 cm (range, 1–10) was performed in 31 femora, and the external fixation index (EFI) was 2 months/cm (range, 0.2–3.1). LLD improved from 4 cm to 1 cm. The most common complication encountered was pin site infection, which was treated with antibiotics. A total of 10 patients developed knee contractures necessitating treatment with quadricepsplasty. Four patients sustained femoral fractures during the treatment course. Complications of fracture were successfully managed with internal fixation in 3 patients and extension of the frame in one patient. One patient in the lengthening group developed acute osteomyelitis which was successfully managed with debridement and intravenous antibiotics. One patient in the bone transport/nonunion group developed chronic osteomyelitis and eventually underwent a successful transfemoral amputation. All other femora are free of infection and have achieved bone union.

### 3.1. Femoral Lengthening Subgroup (Table 2)

**Figure 1:**
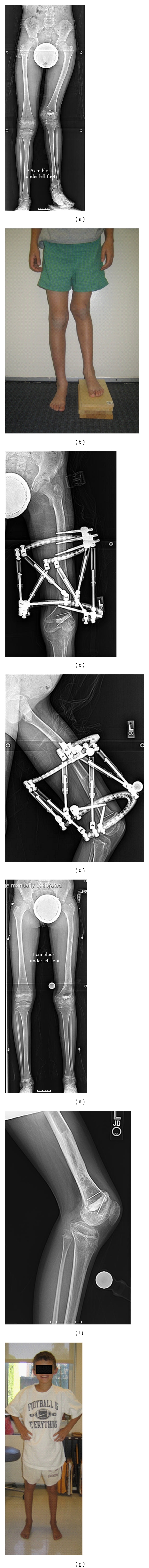
10-year-old boy with left femur growth arrest resulting from a fracture. Prior unsuccessful attempt at bar resection was made. (a, b) Preoperative bipedal radiograph and front view showing valgus deformity and LLD of 6 cm. (c, d) AP and lateral radiographs at end of distraction. An 8 cm lengthening was done with a Taylor spatial frame (Smith and Nephew, Inc., Memphis, TN). Over-lengthening was done in anticipation of additional discrepancy. (e, f, g) Follow-up 6 months after frame removal.

Twenty three femora in 14 male and 7 female patients underwent external fixation for the purposes of lengthening (Table 2). Two patients underwent placement of intramedullary nail after the removal of the frame. Unilateral lengthening was performed in 19 patients, while two patients underwent bilateral lengthening. The etiologies of limb length discrepancy included idiopathic/congenital shortening of femur/lower extremity (six femora), traumatic growth arrest (two femora) (Figure 1), achondroplasia-short stature (2 femora, one patient), growth hormone deficiency-short stature (2 femora, one patient), Klippel-Trenaunay Syndrome (one), Ollier disease (one), status-post femoral fracture (two), femur malunion (two), congenital hip dysplasia (one), hip dysplasia status-post radiation/surgical treatment of lymphoma (one), and polio (one). Associated conditions included rheumatoid arthritis (one patient) and neuropathy (one patient). LLD, varus and valgus deformities were substantially improved with surgery (Table 2).

There were a total of 22 complications for a complication rate of 0.96. The most common complication was knee contracture (7 patients), all incidences of which were treated by quadricepsplasty. No hamstring releases were performed to treat flexion contractures of the knee. There was one case of osteomyelitis which was treated with pin site debridement and IV antibiotics. Six patients developed pin site infections and required a course of oral antibiotics. One patient suffered a subtrochanteric fracture following a fall, which necessitated modification of the Ilizarov frame. There was one case of neurological deficit with the patient developing a foot drop as well as decreased sensation in the foot which resolved over time. Four patients in this subgroup developed bone deformities over the course of treatment. Two patients developed procurvatum deformities. One patient developed a varus deformity which was treated by adjusting the frame. One patient developed a varus deformity of distal femur as well as recurvatum deformity of proximal tibia. 

Ten of the twenty three femora had no additional operative procedures (except for the removal of the fixator). Of the remaining thirteen femora, six femora underwent one additional operation, six femora underwent two, and one femur was operated on four more times. Thus, overall there were 22 additional procedures performed. The procedures performed included quadricepsplasty (seven), gastrosoleus fascial release (one), insertions and removal of intramedullary nail (three), debridement and curettage (three), and iliotibial band release (one).

### 3.2. Femoral Deformities Subgroup (Table 3)

Fourteen femora in seven male and 5 female patients were treated with external fixation for deformities of the femur (Table 3). Unilateral deformity correction was performed in 10 patients, while 2 patients underwent bilateral deformity correction. The deformities included femoral varus (6 patients, 6 femora), femoral valgus (4 patients, 6 femora), flexion deformity of the knee (one patient), and multiapical femur deformity secondary to fibrous dysplasia (one patient). Additional deformities included recurvatum (2 patients), procurvatum (1 patient), and internal rotation (4 patients). Two patients in this subgroup had rheumatoid arthritis, and one patient suffered from lower extremity neuropathy. Diagnosis of diabetes mellitus was present in one patient. 

In 3 of the patients, tibial/fibular osteotomies with application of Ilizarov-Taylor Spatial Frame were performed for the additional deformity correction. Bone grafting was only performed in one patient. Bone stimulation in the form of low-intensity pulsed ultrasound for 20 minutes per day was used in 7 patients.

 Of the 6 patients with varus deformity of the femur, 3 patients had an isolated ipsilateral leg length shortening, one had an ipsilateral limb length shortening with a femur malunion, and one had Blount's disease with ipsilateral limb shortening. Of the 4 patients (6 femora) with femoral valgus deformity, 3 patients (4 femora) had limb shortening. The one patient with flexion deformity of the knee also had an associated limb length discrepancy. 

Of the 12 patients in this group, 9 patients (11 femora) underwent acute deformity correction at the time of surgery, and the frame was only used for the purposes of immobilization and fixation of the osteotomy site. For the 3 patients in whom the frame was used for gradual deformity correction, the average time of correction was 1.49 months. The average total time that all 12 patients spent in the frame was 3.66 months. Just as for patients undergoing lengthening procedures, the patients undergoing deformity correction were also kept in the frame for a certain period of time after the adjustment of the frame was complete to facilitate the healing of the osteotomy site. In this group, the period of fixation averaged 3.23 months (Table 3). 

 Ten patients in this group had LLD. The overall LLD and femoral length discrepancy improved with surgery. Varus and valgus deformities were substantially improved as measured by MAD and LDFA. The range and standard deviation of the LDFA decreased showing that the values were improved toward the normal range. Knee ROM did not change significantly (Table 3).

There were a total of 9 complications in this patient group, for a complication rate of 0.64. The most common complication was pin site infection, which occurred in 3 patients. All of these were successfully treated with Keflex, with supplementary doxycycline used in one case. Knee contracture developed in only 2 patients. It was treated with quadricepsplasty in one patient, while the other underwent both quadricepsplasty and iliotibial band tenotomy. One patient developed peroneal nerve neuropraxia, which was successfully treated with peroneal nerve release.

### 3.3. Bone Transport and Nonunion Repair Subgroup (Table 4)

The third group in our study consisted of patients who had undergone bone transport and nonunion repair to treat femoral nonunion and bone defects (Table 4). In addition to external fixation, two patients in this group underwent placement of the intramedullary nail at the time of surgery with both lengthening and deformity correction being done using both the external fixator and the nail. This group consisted of 10 femora in 10 patients, of whom 8 were male and 2 were female. The application of Ilizarov external fixator or monolateral external fixator was performed unilaterally in all patients. The average patient's age at the time of surgery was 41.50 years.

**Figure 2:**
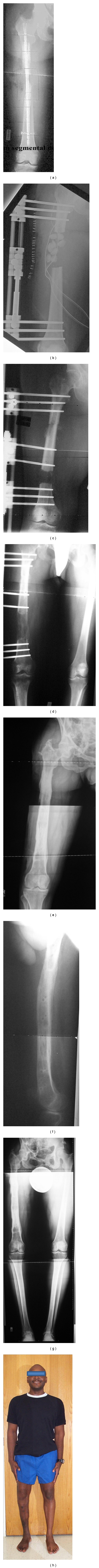
28-year-old male with posttraumatic infected (vancomycin resistant enterococcus fecaelis) femur with 9 cm bone loss. (a) Preoperative AP X-ray showing bone loss and intramedullary rod in place. (b) After stage 1 surgery to remove hardware, apply monolateral external fixator, and insert antibiotic coated cement beads. Gradual shortening of the defect was done. (c) After stage 2 surgery which involved distal femur osteotomy for lengthening and further shortening of the defect. Note the removal of antibiotic beads. (d) At end of distraction. Note 9 cm distal femur lengthening and compression at the proximal docking site. (e)–(h) Follow-up one year after frame removal.

Included in this group were patients with nonunions of variable etiologies, as well as in various stages of treatment. One of the nonunions was infected (Figure 2), while another one was status-post infection with antibiotic beads in place. One nonunion was status-post osteosarcoma resection and reconstruction. One patient's nonunion was above old knee arthrodesis and status-post application of Synthes external fixator following a distal femoral fracture. One patient had a retained intramedullary nail with a segmental area of allograft and devascularized bone.

Femoral deformity was present in one patient. This patient also had an associated femoral length discrepancy as well as having an intramedullary nail in place. Two other patients required bone transports following motor vehicle accidents. One of these patients had a leg length discrepancy with significant sciatic and femoral nerve injuries. The other patient had an infected tibial fracture without length discrepancy of the lower extremities. Two other patients had leg length discrepancies due to bone defects present in the femora. 

Structural deformities in this group included femoral varus (3 femora), internal rotation (2 femora), and external rotation (2 femora). The prevalence of preoperative osteomyelitis in patients undergoing bone transport was greater (4 patients, 40%) than in patients undergoing femoral lengthening (0%) and those with femoral deformities (0%). Three of the patients had neuropathy, with one having significant sciatic and femoral nerve injuries status-post motor vehicle accident and two having diminished sensation on dorsal (1 patient) and plantar (1 patient) foot surfaces. Bone grafting was performed in seven patients. Seven patients utilized bone stimulators (pulsed ultrasound for 20 minutes per day).

The average follow-up after surgery was 53.3 months. Lengthening was performed in 7 patients in this group. The distraction time was 4.27 months. For the rest of the patients, bone defect/nonunion repairs were performed at the time of surgery, and the frame was used solely for the purposes of fixation. The average time of fixation for all 10 patients was 5.61 months. The total time spent in frame was 9.92 months, and the EFI was 1.86 months/cm.

Preoperatively, 8 patients were found to have LLD. In 7 of these, the discrepancy was either totally or partially stemming from the femur. The mean total bone loss in the femur was 7.14 cm. The mean femoral length discrepancy was 5.70 cm. After surgery, the overall LLD improved to 1.71 cm, and the femoral length discrepancy improved to 0.36 cm (Table 4).

Alignment as measured by MAD and LDFA range improved with surgery. A substantial decrease in the knee ROM was noted in this patient group (Table 4). This was principally attributed to the fact that 2/10 patients underwent knee fusion as part of their treatment.

There were 7 complications in this patient group. Two patients developed pin site infection, which were treated with Keflex. One patient developed a wound infection with Methicillin-resistant staphylococcus aureus and required hospital admission for administration of IV antibiotics. Five patients had to undergo additional surgical intervention for modification of their external fixators. Knee arthrodesis was performed in 2 patients. The incidence of knee contracture, treated with quadricepsplasty in this group, was 10% (1 patient). This is a much smaller incidence rate compared to that in patients undergoing femoral lengthening, where the incidence rate was 30%. Three patients developed new deformities over the course of treatment with one case each of varus deformity, internal rotation deformity, and recurvatum deformity. One patient developed chronic osteomyelitis which eventually necessitated a transfemoral amputation. All other patients achieved bony union.

## 4. Discussion (Table 5)

Femoral length discrepancy and deformities have multiple etiologic causes including trauma [15], congenital syndromes [3, 14, 17], metabolic conditions [16], and infections [21] and may lead to osteoarthritis, gait abnormalities, and spinal disorders [8]. The Ilizarov method revolutionized the treatment of these conditions by enabling their simultaneous treatment. The variety of surgical techniques described in the literature makes comparing surgical outcomes across different studies difficult. The unique aspect of this study is its emphasis on the application of the Ilizarov method solely for the operations of the femur. This allows the study to include patients with etiologies spanning a wide spectrum of diseases affecting this particular part of the body. While these patients may share many similarities, the division of the patient population into 3 subgroups enables the authors to focus on aspects of treatment, complications particular to each pathology (Table 5), and facilitates intergroup comparisons. 

One of the indices developed to facilitate comparison of outcomes is EFI, which is the number of months that the patient has spent in the external fixator divided by the cm of lengthening achieved. In our study, the EFI for the lengthening group was 2.15 mo/cm. It must also be noted that the mean age of our patients was 25.29 years and 33.54 years in lengthening and deformity correction groups, respectively. Thus, our average patient was older than patients in all the studies reviewed [1, 3, 5, 15, 17, 20, 23–25] except for one [4]. Given the faster rate of bone growth in younger patients, it makes sense that callus consolidation would take place earlier thus allowing earlier removal of the external fixator in the younger population. Our lengthening experience was comparable to other studies in the literature [1, 3–5, 15, 17, 20, 23–25]. The amount of deformity correction obtained is thus comparable to that reported by Tsuchiya et al. [20]. Gugenheim and Brinker [18] and Nakase et al. [15] report, respectively, higher and lower preoperative severity of the deformities as well as the amount of correction obtained. 

The LDFA averages did not change but the ranges of LDFA became much smaller after treatment. Normal values of LDFA range from 85° to 91°. Values lower than 85° reflect valgus deformity, and values greater than 91° reflect varus deformity. When varus and valgus LDFA values are combined, the average value does not accurately reflect the deformity. The more narrow range of LDFA reflects the improvement in alignment as was seen in all the subgroups.

We have encountered the usual complications common with the Ilizarov external fixator, including pin site infections, decreased knee range of motion, deep infections (osteomyelitis), neurological complications, and new deformities. In the lengthening group, the most common complications were knee contractures, with 7 cases or 30%, requiring quadricepsplasty. This complication has been noted by multiple authors [1, 4, 5, 15, 20, 24, 25], with rates ranging from 0% to 100%. Quadricepsplasty rates ranged from 0% to 18% in series utilizing external fixation  ±  intramedullary nail [1, 5, 15, 20] and 0% in series using intramedullary nails only [4, 24, 25]. This is consistent with the rates reported in the literature which range from 16.7% to 90% [1, 3, 5, 15, 20]. There were 3 patients who developed deformities in the sagittal plane (2 procurvatum and 1 recurvatum). This complication has previously been reported by Iobst and Dahl [1] (33% incidence) and Nakase et al. [15] (14.3%). There was one case of fracture of the callus secondary to a fall (4.3%). This is significantly less than the rates previously reported (7.4%–16%) [1, 15, 17, 20]. In the deformity correction group, pin site infections were the most common complication (3 cases or 21%). Two cases of knee contractures requiring quadricepsplasty were noted in this group (14%). Previously, the rate of quadricepsplasty in patients undergoing external fixation only for the purpose of deformity correction has been reported as 15% [26]. One patient developed septic arthritis of the knee. There was one case of peroneal nerve neuropraxia. This complication was also noted by García-Cimbrelo et al. [25]. No new deformities developed in this group. 

 Prior to the development of the Ilizarov method, the treatment of bone loss and nonunions caused by traumatic injuries or infections involved the use of cancellous autogenous bone grafts, vascularized autografts, structural allografts, and artificial bone substitutes as well as internal fixation [6, 8]. The treatment of nonunions using the Ilizarov method depends on the type of nonunion. In hypertrophic nonunions, which have sufficient vascularity to promote bone healing but lack the necessary structural stability, Ilizarov advocated gradual compression of the two surfaces of the nonunion to promote consolidation, followed by gradual distraction to compensate for the associated bone loss. In atrophic nonunions, which have significantly diminished bone healing capacity due to decreased vascularity, compression and distraction are carried out simultaneously at different sites in the bone. The bone is compressed at the site of the nonunion. A separate osteotomy is performed at another part of bone fragment, and distraction is performed there. The two components of treatment, namely, achievement of union and lengthening may also be separated in time. Indeed, many patients opt to have one surgery to achieve union and then come back later to undergo a second surgery for the treatment of length discrepancy and deformities secondary to nonunion [7, 22]. This is due to the prolonged length of treatment and limitation of activities of daily living during its course due to the bulk of the external fixator. 

In our study, the group undergoing external fixation with the primary goal of bone transport/nonunion had a mean external fixation index of 1.86 mo/cm. This is comparable to the values reported previously, except for Krishnan et al. [9]. There is a significant difference in EFI reported between series where external fixation was used alone [6–9, 11, 27] versus those where it was combined with intramedullary nailing [8, 10]. This difference was very well demonstrated by Zhang et al. [8], who compared these two groups within the same study. The complications encountered in our patient population were consistent with previously published ones. The most common complications encountered included pain during the distraction phase, pin-tract infections, and decreased range of motion at the knee joint. The rate of pin-site infection in our study for the patients undergoing treatment of nonunion/bone transport was 20%. This was lower than the rates previously reported [6, 8–10, 28] and can be attributed to the meticulous pin-site wound care. The rates of amputation, osteomyelitis, neurovascular complications, and fracture after external fixator removal were comparable with the literature [6–11, 27–30]. It has been noted [28] that femoral nonunions may be associated with shortening due to bone resorption at the fracture site, which can be further exacerbated with compression using the external fixator. Indeed, both Inan et al. [28] and Brinker and O'Connor [30] discuss treatment outcomes where the limb length discrepancy was increased following treatment. We did not encounter this complication in our patient population. Knee ROM was noted to decrease in only the nonunion/bone defect group. This can be attributed to longer times in the frame than the other groups and the fact that 2 of the 10 patients underwent knee arthrodesis.

 The results of our study indicate that external fixation is an effective method for the treatment of femoral length discrepancies, deformities, and nonunions secondary to multiple etiologies. Some of the advantages of the technique include shorter operating times, decreased blood loss, decreased surgical exposure, preservation of blood supply to bone which facilitates bone healing, enhanced mechanical stability, which allows early ambulation, and the ability to perform simultaneous lengthening and deformity correction [1, 6, 22]. As noted by multiple authors [4, 22, 26, 31–34], this technique while effective does carry with it some distinct complications including pin site infections, pain during lengthening, neurovascular damage secondary to pin insertion, muscle contractures, joint stiffness and dislocations, and refracture of the callus site secondary to inadequate external fixator stability or after fixator removal. The frame is also bulky and, while facilitating early ambulation, may nonetheless prevent full patient participation in activities of daily living. The need for multiple daily precise distraction adjustments as well as for meticulous pin site care also impairs the quality of life of patients while utilizing the external fixator. One of the recent developments to decrease the time that patients spend in the frame is the combined use of external fixator and internal fixation, such as an intramedullary nail. These can be used sequentially (lengthening and then nailing) [35] or simultaneously (lengthening over the nail) [5]. These approaches eliminate the bulky frame while at the same time protecting the still unstable callus site from fracture. As has been noted above, the time that the patients spend in the external fixator is significantly decreased with these combination methods. It must be noted again that the discrepancy in results between various studies is attributable to multiple factors including the differences in external fixators produced by different manufacturers, surgical technique, patient populations and etiologies, and the utilization of accessory devices such as intramedullary nails and submuscular plates in some studies, but not in others. Despite the disadvantages mentioned above, the Ilizarov method with the use of the external fixator, as well as its latter modifications, provides surgeons with a comprehensive approach to multiple femur pathologies and by its inherent adaptability to various clinical situations can be expected to continue to play important roles in the future. It is particularly useful in the setting of bone loss, LLD, infection, large and complex deformity, and poor soft-tissue envelope.

## Figures and Tables

**Table 1:** Results for entire group.

Parameter	Preoperative	Postoperative
LLD (cm)	4 (range, 0–17)	1 (range, 0–9)
Varus deformity (mm medial MAD)	36 (range, 6–100)	9 (range, 0–44)
Valgus deformity (mm lateral MAD)	24 (range, 7–57)	8 (range, 0–43)
LDFA (deg.)	89° (SD 8.4)	89° (SD 4.6)
Knee ROM (deg.)	0° (SD 7.9)–118° (SD 33.3)	1° (SD 2.8)–106° (SD 39.7)

LLD: leg length discrepancy; ROM: range of motion; deg.: degrees; LDFA: lateral distal femoral angle; MAD: mechanical axis deviation.

**Table 2:** Femoral Lengthening data.

Number of patients	21
Number of femora	23
Age (years)	25 (range: 9–57)
Preoperative deformities	Varus (4), valgus (7), procurvatum (2), internal rotation (2)
Preop medical conditions	Rheumatoid arthritis (1), neuropathy (2)
Follow-up time after surgery (months)	38.9 (range: 7.3–70.0)
Preoperative varus (mm medial MAD)	12.64 (range: 0–35)
**Post-operative varus (mm medial MAD)**	**7.0** **(range: 0–20)**
Pre-operative valgus (mm lateral MAD)	16.73 (range: 0–36)
**Post-operative valgus (mm lateral MAD)**	**11.29 (range: 0–43)**
Pre-operative LDFA (deg.)	87.57 (range: 74–102)
**Post-operative LFDA (deg.)**	**88.77 (range: 81–98)**
Pre-operative knee ROM (deg.)	−0.24° (SD 4.9) –132° (SD 4.2)
**Post-operative knee ROM (deg.)**	**0.22° (SD 1.0) –123° (SD 12.2)**
Preoperative total LLD (cm)	4.7 (range: 2.0–12.6)
Preoperative femoral length discrepancy (cm)	2.98 (range: 0.8–8.0)
**Postoperative total LLD (cm)**	**1.29 (range: 0–8.5)**
**Postoperative femoral length discrepancy (cm)**	**0.30 (range: 0–2.7)**
Number of patients requiring bone graft	3
Distraction time (months)	1.9 (range: 0.5– 5.1)
Time of consolidation (months)	3.87 (range: 0–9.2)
Total time in frame (months)	5.78 (range: 0.69–12.1)
External fixation index (months/cm)	2.15
Complications	Knee contracture (7), pin site infections (6), procurvatum (2), varus (2), osteomyelitis (1), foot drop (1), subtrochanteric fracture secondary to fall (1), recurvatum deformity of proximal tibia (1), ankle equinus contracture (1)

LLD: leg length discrepancy; ROM: range of motion; deg.: degrees; LDFA: lateral distal femoral angle; MAD: mechanical axis deviation; postoperative values in bold.

**Table 3:** Femoral deformity correction data.

Number of patients	12
Number of femora	14
Age (years)	33.5 (range: 21–49)
Follow-up time after surgery (months)	35.8 (range: 8.1–49.7)
Pre-operative deformities	Varus (6), valgus (6), recurvatum (2), procurvatum (1), internal rotation (4)
Pre-operative medical conditions	Diabetes (1), rheumatoid arthritis (2), neuropathy (1)
Pre-operative varus (mm medial MAD)	47.8 (range: 18–100)
**Post-operative varus (mm medial MAD)**	**11.1 **(range: 0–25)
Pre-operative valgus (mm lateral MAD)	27.7 (range: 8–57)
**Post-operative valgus (mm lateral MAD)**	**4.8 **(range: 0–9)
Pre-operative LDFA (deg.)	88.6 (range: 80–114)
**Post-operative LFDA (deg.)**	**91.1 **(range: 84–105)
Pre-operative knee range of motion (deg.)	0.71° (SD 12.4) –120° (SD 18.7)
**Post-operative knee range of motion (deg.)**	−2.0° (SD 4.6) –116.8° (SD 20.2)
Pre-operative total LLD (cm)	2.1 (range, 0.4–8.3)
**Post-operative total LLD (cm)**	**1.4 **(range: 0–7.4; SD: 2.26)
Pre-operative femoral length discrepancy (cm)	1.5 (range: 0.4–4.7)
**Post-operative femoral length discrepancy (cm)**	**0.7** (range: 0–3.7; SD: 1.35)
Number of patients requiring bone graft	1
Distraction time (months)	1.5 (range, 0.26–2.30; SD: 0.88)
Consolidation time (months)	3.2 (range, 2.0–7.1; SD: 1.33)
Total time in frame (months)	3.66 (range, 2.5–7.7; SD: 1.42)
External fixation index (months/cm)	
Complications	Pin site infections (3), knee contracture (2), peroneal nerve neuropraxia (1), septic arthritis of knee (1)

LLD: leg length discrepancy; ROM: range of motion; deg.: degrees; LDFA: lateral distal femoral angle; MAD: mechanical axis deviation; postoperative values in bold.

**Table 4:** Bone transport and nonunion repair.

Number of patients	10
Number of femora	10
Age (years)	41.5 (range: 11–89)
Follow-up time after surgery (months)	53.0 (range: 13.6–75.7)
Pre-operative deformities	Varus (3), internal rotation (2), external rotation (2)
Pre-operative medical conditions	Osteomyelitis (4), neuropathy (3), diabetes (1)
Pre-operative varus (mm medial MAD)	36.8 (range 0–64)
**Post-operative varus (mm medial MAD)**	**11.3** (range: 0–44)
Pre-operative valgus (mm lateral MAD)	7.0 (range: 0–14.0)
**Post-operative valgus (mm lateral MAD)**	**3.2** (0–9.0)
Pre-operative LDFA (deg.)	94.0 (range: 79–106)
**Post-operative LFDA (deg.)**	**85.9** (range: 81–92)
Pre-operative knee range of motion (deg.)	0.0° (SD: 0) –71.43° (SD: 60.67)
**Post-operative knee range of motion (deg.)**	0.0° (SD: 0) –46.67° (SD: 52.97)
Preoperative total bone loss (cm)	7.1 (range: 0.0–16.5)
**Post-operative total LLD (cm)**	**1.7** (range: 0.0–5.0)
Pre-operative femoral length discrepancy (cm)	5.7 (0.0–16.5)
**Post-operative femoral length discrepancy (cm)**	**0.4** (range: 0.0–1.4)
Number of patients requiring bone graft	7
Distraction time (months)	4.3 (range: 0.7–6.7; SD: 1.97)
Time of fixation (months)	5.6 (range: 0–12.5, SD: 4.1)
Total time in frame (months)	9.9 (range: 0.95–17.8)
External fixation index (months/cm)	1.86
Complications	Pin site infection (2), chronic osteomyelitis leading to amputation (1), knee contracture (1), internal rotation (1), varus (1), recurvatum (1)

LLD: leg length discrepancy; ROM: range of motion; deg.: degrees; LDFA: lateral distal femoral angle; MAD: mechanical axis deviation; postoperative values in bold.

**Table 5:** Subgroup Comparison table.

	Femoral lengthening	Femoral deformities	Nonunion correction/bone transport
Number of patients	21	12	10
Number of femora	23	14	10
Age	25.29	33.54	41.5
Follow-up time after surgery (months)	38.9	35.8	53.03
Pre-operative varus (mm medial MAD)	12.64	47.83	36.75
**Post-operative varus (mm medial)**	**7.0**	**11.09**	**11.29**
Pre-operative valgus (mm lateral MAD)	16.73	27.71	7.0
**Post-operative valgus (mm lateral)**	**11.29**	**4.75**	**3.20**
Pre-operative LDFA (deg.)	87.57	88.57	94.0
**Post-operative LFDA (deg.)**	**88.77**	**91.14**	**85.9**
Pre-operative knee ROM (deg.)	−0.24–131.82	0.71–120	0.0–71.43
**Post-operative knee ROM (deg.)**	**0.22**–**123.04**	**−2.0**–**116.79**	**0.0**–**46.67**
Total pre-operative LLD/bone loss (cm)	4.69	2.14	7.14
**Post-operative total LLD (cm)**	**1.29**	**1.36**	**1.71**
Total pre-operative femoral leg length discrepancy (cm)	2.98	1.46	5.70
**Post-operative femoral length discrepancy (cm)**	**0.30**	**0.72**	**0.36**
Distraction time (months)	1.92	0.46	4.27
Consolidation (months)	3.87	3.23	5.61
Total time in frame (months)	5.77	3.66	9.92
External fixation index (months/cm)	2.15		1.86
Complications	22	9	7

LLD: leg length discrepancy; ROM: range of motion; deg.: degrees; LDFA: lateral distal femoral angle; MAD: mechanical axis deviation; postoperative values in bold.
